# Modern Medico-Psychology and Psychiatry

**Published:** 1894-05-26

**Authors:** T. S. Clouston

**Affiliations:** Lecturer on Mental Diseases, Edinburgh University, and Physician Superintendent Royal Asylum, Morningside


### Modern Medico-Psychology and Psychiatry.

By T. S. Clouston, M.D., Lecturer on Mental Diseases, Edinburgh University, and Physician Superintendent Royal Asylum, Morningside.

### VI.—THE MEN WHO HAVE MADE MODERN PSYCHIATRY (*continued*).

The architecture of the brain is so infinitely complicated, and its structures so delicate, minute, and difficult to manipulate and unravel, that it has taken the utmost mechanical ingenuity of some of our best histologists for thirty years to get as far as we are now. I am old enough to remember the time when the present most fruitful methods of hardening, and latterly of freezing, the delicate jelly-like structure of the brain had not been invented, when the methods of cutting the hardened mass into thin sections were unknown, and when the clearing up of those tenuous sections so that the structures they contained could be seen by high powers of the microscope, had not been thought of. The staining of these hardened sections by long steeping in solution of carmine so as tc bring out the cells and fibres as at first practised, the improvements on this by Bevan Lewis's aniline blue black stain, the Weigert methods, and the last and most vivid method of Golgi, whereby each brain cell and fibre is incrusted by a thin film of silver deposited on it, and stands out like the black letter type of an old book under the microscope, have all come out in the lifetime of men now only in mid-age. I smile inwardly whenever I think of the late Professor Goodsir, of Edinburgh, one of the greatest histologists of his time, going over with me in 1861 my specimens from the nervous ganglia of lobsters and crabs that I had hardened in absolute alcohol, and cut into sections while held as best I could in my fingers, and then "cleared up" with a solution of chloride of calcium, and his pronouncing some of them to be one of the finest demonstrations of nerve cells he had ever seen, and my getting a gold medal for my graduation thesis for such crude work. From these to the cells, as seen by Golgi's method that we now prepare here, seems a leap from brain darkness into noonday as regards our knowledge of the brain cell, and yet I know full well that as much or more remains still to be discovered as to the structure, appearance, groupings, and relations of those million nerve batteries which each man's brain contains. A brain cell in 1860 was to our conception, a small bladder with one or two necks to it; now it is a minute living battery with charging and discharging apparatus, with an infinitely delicate framework and innumerable connections with and relations to other similar batteries all as complicated as itself.

The magnificent discovery of Hitzig, and Fritz that the application of electric currents to different parts of the cortex of the brain produced movements in the extremities of the body according to the part so stimulated, and the fruitful application of this "localisation" discovery by Ferrier has revolutionised our ideas as to the mental working of the brain in health and disease. No longer can we hold to the old vague theory that mental operations reside equally in every part of the brain, unlocalised and formless. We now know through these discoveries that every brain convolution has its own special function, and every cell its definite work. As the problems of definite fibres for every ingoing impression on the senses, of definite cells in the brain set apart to receive these impressions, to register them and to revivify them on suitable stimulus; of the relationship of such impressions to the ten thousand other impressions that have travelled inwards to other brain cells and been registered every year of a man's life; of the connections and co-relations of all such impressions to each other, and to the sensations, the perceptions, and the concepts they arouse, and the thoughts, imaginations, memories and feelings that we know do arise in consequence of the original sense impressions—as all these problems are attempted to be grasped, a sense of bewilderment falls on one's mind. The mighty climax to the process of organic evolution through countless ages that we find in the human brain, as revealed to us by the modern physiologist, stuns us by its ordered complexity, just as our minds are affected by gazing up at the stars on a dark night, and thinking about what they mean. If a human brain could be magnified ten thousand fold, so that we could see and realise its millions of arranged and classified cells and fibres, its protective, nourishing and its draining apparatus, it would be seen to be as a mere apparatus the most wonderful and the most complicated in nature. A modern cotton mill would be simplicity itself compared to it. If we saw this apparatus at work, no human imagination could conceive how its multitude of interdependent parts could possibly work harmoniously. Responsive to every force in nature, governing the world through its energies, always active, retaining from birth to death the results of countless impressions from within and from without this is the organ whose disturbances the medical psychologist has to study, to endeavour to understand, and to restore to normal working if he can. The task is assuredly so difficult a one that many hundreds of years of study will not see it fully accomplished. But the mental physician of to-day must as a basis of all his conceptions of insanity and all his treatment of it, found on the work of the cerebral physiologist and histologist. That work must be consciously referred to in every hypothesis he forms and every conclusion he comes to. Some men, like Meynert and Bevan Lewis, are profound and original physiologists and histologists as well as being advanced psychiatrists.

The fourth and chief group of men that have advanced modern psychiatry have been the great mental physicians of this century, some of them working chiefly as physicians pure and simple, and some of them working towards the advancement of the treatment of the insane through asylum administration. The two modes cannot be separated. To study a disease clinically, symptom by symptom, case by case, to co-relate one observation with another, to generalise and attempt to formulate laws on the co-related facts, to treat by drugs and diet, to condition favourably the lives of the patients by a suitable administration of a curative hospital so as to antagonise the disease, are all essential parts of a true mental physician's work. None of them can be left undone without some amount of failure resulting. Almost all the Europern countries and America have produced great mental physicians, but France must be placed first on the list as having produced Pinel, Esquirol, and Bayle late in the last and early in this century. Pinel studied and classified insanity and cerebral pathology after having taken the fetters off the insane at the Bicêtre. Esquirol further classified, fully described, and attempted to condition and so cure insanity, embodying his results in his great work, "Maladies Mentales," and instituted, regulated, and described the then model hospital for the insane at Charenton. Bayle discovered general paralysis, and so put the study of mental diseases once for all on a pathological basis. These men have had great successors in Morel, Moreau, Baillarger, Foville, Falret, Brierre de Boismont, Dagonet, Motet, Ball, Voisin, Charcot and Féré, and many more.

